# Health Education and Vaccination for the Construction of Inclusive Societies

**DOI:** 10.3390/vaccines9080813

**Published:** 2021-07-22

**Authors:** Eduardo García-Toledano, Ascensión Palomares-Ruiz, Antonio Cebrián-Martínez, Emilio López-Parra

**Affiliations:** 1 Department Public health, Childhood Cancer Foundation, 28029 Madrid, Spain; Toledanoeg@gmail.com; 2 Department of Pedagogy, Universidad de Castilla-La Mancha, 02071 Albacete, Spain; Antonio.Cebrian@uclm.es (A.C.-M.); Emilio.LopezParra@uclm.es (E.L.-P.)

**Keywords:** vaccines, public health, vaccine reluctance, education, inclusive societies, COVID-19, immunization, bootstrap, gender

## Abstract

Globalization has led to what has happened in a certain part of the world having a significant and rapid impact on other places, causing significant changes in health problems. In the last quarter of the 18th century, the history of vaccination began, becoming an effective tool to prevent and control communicable diseases. This paper proposes an observational research with a cross-sectional design to study the importance of health education and vaccination in building inclusive societies. With a sample of 1000 participants from 76 countries, vaccine awareness and regulation were analyzed, considering the following variables: gender, age, sector, Human Development Index (HDI), and continent. The instrument used was a questionnaire (VACUNASEDUCA) developed for this research and timely validated. As a result, it is highlighted that the profiles of women, people under 30 years of age, education sector, high Human Development Index, and European continent are those that most value the importance of raising awareness in society and the regulation of actions for vaccination compliance. The consequences of “vaccine reluctance” are of concern in every country on the planet. Therefore, it is concluded that effective and evidence-based communication is key to allaying fears and promoting acceptance of vaccination around the world, building inclusive societies in which all citizens enjoy the health benefits.

## 1. Introduction

In recent decades, globalization has caused events, decisions, and activities happening in each place on the planet to have a significant and rapid impact elsewhere in the world. All this leads to a significant change in health problems and societal concerns. Reasonably, many difficulties have their origins in social factors that cause inequalities in health, but it should be noted that it is the growing socioeconomic inequalities that generate the greatest inequalities in health, so that poverty is the main socioeconomic factor that affects the state of health of people [1]. Indeed, the world is immersed in various challenges, one of the most significant being the effects of the new technological revolution that is generating the transformation of humanity. Therefore, the concept of the “fourth industrial revolution” arises within the framework of the World Economic Forum to refer to changes in the global economy from the basis of technologies supported by physical systems, biological systems, digital systems, and their interactions, generating a fundamental change in the way we live, work, and relate [2].

It should be recalled that, in 1789, Edward Jenner was the first to scientifically demonstrate that people could be protected against smallpox, if exposed to smallpox vaccine [3,4]. However, it should be noted that, a few years earlier, in 1774, there is a reference to the first person who performed a vaccination; in this case, he vaccinated his family with smallpox vaccine [5]. Nowadays, vaccines can prevent and control communicable diseases and are considered essential to deal with emerging infectious diseases; they can immobilize or limit epidemic outbreaks of these, and combat the spread of antimicrobial resistance. For this reason, society is demanding that the industry and the scientific community respond with vaccines, as soon as possible, to the epidemics of H1N1 flu, Ebola, Zika, COVID-19, etc. [6].

As it is known, vaccines are antigenic preparations made up by micro-organisms, or by part of these, which are modified to lose or attenuate their pathogenic capacity, and their purpose is to stimulate the individual’s defence mechanisms against infectious agents. Advances in molecular biology, specifically in the development of recombinant DNA technology and bioinformatics, have been essential for the evolution of vaccines [7]. Consequently, diseases such as smallpox have been eradicated globally, and other diseases, such as polio and measles, have been significantly reduced, as have the disabilities and deaths these diseases can cause. Its effectiveness is evidenced by the fact that, when immunization coverage decreases, diseases that have not been completely eradicated rebound, such as measles in Europe in recent years [8] or in the United States, which, in 2019, reported the highest number of cases since 1992 [9]. Immunization also plays a key role in achieving UN Sustainable Development Goals (SDG), specifically SDG3, “Ensuring healthy living and promoting well-being for all at all ages”, and contributes to the achievement of the other 16 SDGs [10].

It should be noted that there is no 100% effective vaccine, nor are they all equally effective; however, the benefits of vaccines focus on their proven effectiveness, which is revealed in their behavior in practice and depends, fundamentally, on the immune capacity of the recipient, the type of vaccine, established schedules, availability, tolerance, and stability.

Since the creation of the Expanded Immunization Program by the World Health Organization (WHO) in 1974, benefits have been expanded and there is explicit recognition of the importance of vaccination and its high impact on social welfare. Global Vaccine Action Plan 2011–2020 (GVAP) [11] was approved by 194 countries in the World Health Assembly. Consequently, the evaluation of GVAP results in 2019 [12] facilitated the development of new proposals until 2030. It should be noted that the GVAP increased the visibility of immunization and assisted in the creation of a high-level political will. Indeed, it created a common framework for prioritizing, agreeing on activities, and evaluating results. The Immunization Agenda (AI2030) [12] has designed a comprehensive global vaccine and immunization strategy for the decade 2021–2030. Reasonably, AI2030 takes advantage of the lessons derived from GVAP and considers the new and persistent challenges posed by infectious diseases.

The European Union (EU) carries out public health policies, but there is no common prevention model. This situation creates inequality to access to immunization among entire communities of citizens, as evidenced by the COVID-19 pandemic. It should be noted that each State has specific legislation on vaccination. Although each country’s vaccination plans are similar, there are significant differences in the vaccines included in the publicly funded vaccination schedules. In addition, the same vaccines are not given and not all vaccines are mandatory, as in Spain, where, to date, they are all voluntary in nature. Each country designs its vaccination strategy with respect to its epidemiological situation; however, WHO, the EU, and United Nations International Children’s Emergency Fund (UNICEF) develop global and European prevention and immunization strategies and plans [13].

Consequently, the AI2030 [12] aims to strengthen existing partnerships, establish new relationships, and better clarify roles and responsibilities in order to improve the use of information to drive action and ensure relevant assessments.

Vaccines are an excellent investment generating an improvement in quality of life, as demonstrated by the Decade of Vaccine Economics project [14], which has been most evident in the most disadvantaged countries. In addition, as González-García points out [15], more investment would have to be made in research and development, which will enable humanity to face with hope and strength any biological threats and pandemics that are to come, which would mean an excellent investment in the future, thus improving the quality of life around the world.

WHO [16] considers vaccination, along with “clean water”, as one of the biggest public health successes, and scientific evidence has shown that the benefits of vaccination outweigh the unusual adverse effects. However, since the onset of vaccines, out of fear or carelessness, there are detractors of these, but the fashion of not vaccinating and the anti-vaccine movement began to grow more and more from 1998 onwards, when a doctor, Andrew Wakefield, presented in the scientific journal *The Lancet* a study linking autism with the triple viral vaccine (measles, rubella, and mumps). The study was based on a very small sample, biased, and without any scientific rigor. As a result of this article, other studies were conducted with different results, demonstrating that it had been a scientific fraud [17].

Currently, rejection of vaccination is occupying large spaces for debate and discussion in the media and social media, in the various areas of society, and in all countries of the world. There are concerns about how the problem is being raised as, in most cases, it is reduced to the polarization between pro-vaccines and anti-vaccines without seriously addressing the issue of vaccination considering the importance of group immunity and collective health. Vaccination reluctance ranges from acceptance to total rejection, with different nuances called “vaccine reluctance” [18]. For years, the consolidation of vaccine rejection has been rated as a growing threat to collective health [19].

The recommendations proposed by WHO [20] focus on the need for a better awareness of vaccination reluctance, its determinants, and the challenges it poses. It also stresses the need to improve society’s awareness of the importance of vaccination in order to improve vaccine acceptance, share effective practices, and develop new tools to assess and address reluctance. Reasonably, in order to improve society’s awareness, it is essential to analyze the benefits and effectiveness of vaccines, concerns about their safety, and how they are perceived in society. Indeed, as Matesanz points out [21], having an effective vaccine is not an individual solution, but that it is received by the maximum number of people in the environment to achieve the long-awaited “herd immunity” and that the virus stops circulating.

It should be emphasized that the information on COVID-19 is overflowing us and relegating to the background the importance of other vaccines that are fundamental to children, especially for the most vulnerable countries. In addition, as García-Toledano [22] points out, the problem with vaccination programs is that they are linked to constant variations linked to vaccine availability, distribution, and administration logistics. Therefore, incorrect communication and co-ordination can show society a negative image of improvisation and lack of co-ordination that do not benefit awareness of the importance of vaccination.

It should be noted that, possibly, in the absence of regulation by administrations over the past two decades, the number of parents who choose not to vaccinate their children has increased. This increase can also be affected because the propaganda that opposition groups broadcast today has much more advanced and powerful information transmission tools every day, through social networks that facilitate the distribution of their opinion to a large number of people in a short period of time. In addition, some public figures publicly support this non-vaccination movement, leading to greater confusion in the population. Fortunately, the Spanish population is generally highly aware of vaccination, reaching immunization rates of 96% in childhood. This figure has fallen slightly in recent years, but it may not be as a result of these opposition associations, but also for socioeconomic reasons in certain marginal groups, so it is important to carry out educational projects aimed at these sectors of the population [22].

If we focus on the current situation caused by COVID-19, WHO [20] has stated that, although coronavirus vaccination has intensified, the world will not achieve collective immunity in 2021. For this reason, internationally renowned organizations, such as the United Nations Educational, Scientific, and Cultural Organization (UNESCO), WHO, UNICEF, the Council of Europe, the Organization for Economic Co-operation and Development (OECD), and the European Commission, indicate that it is essential that schools incorporate health education into their curriculums as a basic tool for developing healthy lifestyle habits, increasing the quality of life of schoolchildren and, consequently, working on building a better world [23].

In the context we find ourselves, our main purpose is to study the importance of education for health and vaccination, analyze its development, and assess its situation in the 21st century.

## 2. Materials and Methods

An observational research was performed using a cross-sectional design which focuses on the study of health education and vaccination for the construction of inclusive societies. The awareness and regulation of vaccines (Dimension 1) were, therefore, analyzed with the aim of responding to problems: P1, society awareness of the importance of vaccination; P2, regulation of administrations of actions for vaccine compliance.

### 2.1. Population and Sample

The sample consists of 1000 participants from 76 countries. The distribution of the participating sample is defined in the results section of the investigation.

**Figure 1:**
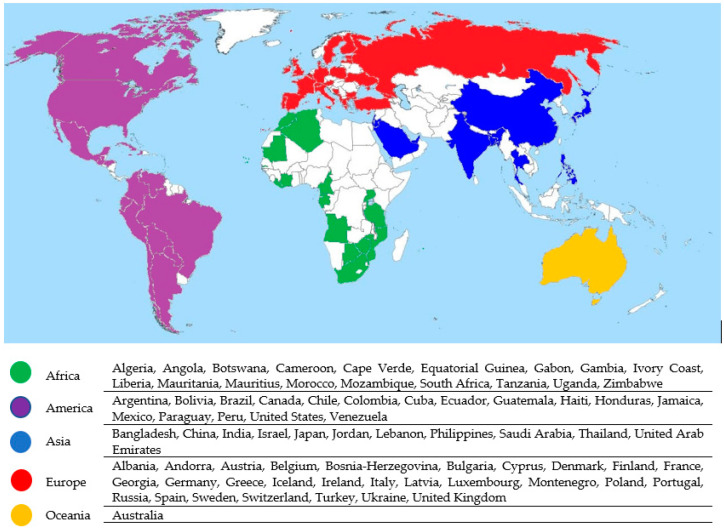
Nationality of study participants. Source: own elaboration.

The study population is delimited based on the selection criteria of developing their professional activity in one of the three sectors analyzed: health, education, and economics. All persons with a job performance not directly linked to these sectors were excluded. The sample selection followed a non-probabilistic sampling method of an accidental or consecutive type, consisting of 1000 participants from 76 countries (Figure 1).

### 2.2. Variables

The questionnaire used VACUNASEDUCA [24] consists of 12 items: 2 items corresponding to dimension *D1 = Awareness and regulation,* 4 items corresponding to dimension *D2 = Education and teachers,* 2 items corresponding to dimension *D3 = Regulation* and obligation, and 4 items corresponding to dimension *D4 = Consequences and risks*.

As an answer option for each item, a Likert scale of 1 to 3 was raised indicating the degree of agreement of the participant with the corresponding question; in this way, there were 12 ordinal variables for each of the items in the questionnaire.

The dependent variable S3t was built by adding the individual ordinal scores for each of the participants and dividing by 12 to typify it; in this way, we could have a quantitative dependent variable that came to represent the degree of agreement of each participant with the 12 items of the questionnaire that would allow us to perform the inferential analysis for each of the independent variables or factors of the research:

- Gender: independent dichotomic variable with 2 *options, G0 = Woman or G1 = Man*.
- Age: polyatomic independent variable with 4 *options, E1 = Less than 30*, *E2 = Between 30 and 44*, *E3 = Between 45 and 59*, or *E4 = Greater than 60*.
- Sector: polyatomic independent variable with 3 *options, S1 = Health, S2 = Education, or S3 = Economy*.
- Human Development Index (HDI): polyatomic independent variable with 4 *options, I1 = very high, I2 = high, I3 = medium, or I4 = low*.
- Continent: polyatomic independent variable with 5 *options, C1 = Europe, C2 = America, C3 = Asia, C4 = Africa, or C5 = Oceania*.

### 2.3. Instrument

The technique used for data collection was the survey, the instrument chosen being a questionnaire consisting of 12 items with a Likert scale.

The questionnaire was developed for the purpose of this research, being submitted to a trial by 15 experts, which allowed Lawshe’s content validity index (CVI) [25] to be calculated, which suggested a CVI 0.51 when using 14 experts, so there was no need to remove any items from the initial questionnaire. Table 1 reflects the CVI for each of the dimensions of the questionnaire, the mean index being 0.96 [26,27,28].

The questionnaire was also validated through exploratory factor analysis. The result of the Kaiser–Meyer–Olkin KMO test was 0.784, revealing the sample adequacy for performing factor analysis. The result of Bartlett’s sphericity test yielded a significance level of 0.000, which involved suitability for factor analysis. Although the matrix of factorial analysis components did not come out completely “clean”, with 2 items appearing correlated with 2 factors, the factorial structure obtained was very similar to that initially designed by García Perales et al. [28] and Méndez & Rondón [29].

The reliability of the questionnaire, in the sense of stability of the results, was calculated through the alpha coefficient of Cronbach (α). Table 1 shows the indexes obtained for each of the dimensions. The mean value for the 4 dimensions was 0.64, being close to the 0.70 limit that Kerlinger et al. [30] set for acceptable consistency.

### 2.4. Procedure

The questionnaire was prepared between June and July 2019 and was implemented from September 2019 to March 2020, specifically, of September to December at the headquarters of the WHO Geneva, and from January to March in Spain, in hospitals, universities, International Congresses of Education, and Congresses and meetings of Medicine. 

The questionnaire was completed in a self-administered form, on paper and in person, in two languages, English and Spanish. There was no time limit, such as the respondents usually would take between 5 and 10 min, and the pollster was always the same person. The research was conducted at the beginning of the COVID-19 pandemic and was not affected by confinement. Anonymity and confidentiality of participants’ data was guaranteed at all times.

### 2.5. Data Analisys

In view of noncompliance with the premises of parametric methods, since the sample distribution did not conform to the normal distribution, it was chosen to use statistical techniques of null models using resampling techniques through the Monte Carlo simulation method using the bootstrap procedure [31], which can be included within the approach of resampling data that perform computer simulation processes that are based on the extraction of a large number of repeated samples from the data themselves, and on which descriptive and inferential statistical analysis is performed using confidence intervals (ICs) extracted from the data themselves. This is what distinguishes such new procedures from classical statistical techniques that are based on theoretical models developed analytically. Consequently, some authors describe these techniques as intensive computation methods and are included within a modern statistical approach, competing with the classical mathematical approach [32,33]. This new approach is based on the enormous computing capacity of modern computers and the sufficiency of the sample to represent or reflect relevant aspects of the population from which it was extracted, since bootstrap’s method allows “the maximum from the little information available” to be extracted [34] (p.149).

To analyze whether there were statistically significant differences, an ANOVA test was chosen for independent samples for each of the independent variables or factors in the research. The values of the F statistic, significance level p, and effect size measured by eta squared were obtained using the analysis of the multivariate general linear model of the SPSS statistical program in version 26. Post hoc tests were conducted assuming not equal variances using Tamhane’s T2, Dunnett’s T3, Dunnett’s Games–Howell, and Dunnett’s C statistics, all of which yielded similar results that served to determine the direction column in the ANOVA tables for each of the factors analyzed.

## 3. Results

First, Table 2 shows the main characteristics of the sample in relation to gender, age, HDI, sector. and continent.

In order to contextualize Dimension 1 in the research on the importance of health education, Table 3 shows the descriptive statistics obtained in the four dimensions of the questionnaire used.

In Table 3, you can see high results for dimensions *D1 = Awareness and regulation* and *D2 = Education and teachers*, *highlighting* item P06 that gets the upper mean score (M = 2.86, SD = 0.44), the highest mean value of the instrument set, and low results *for dimensions D3 = Regulation* and obligation *and D4 = Consequences and risks* by highlighting items P11 and P12 that get the lowest mean score (M = 1.18, SD = 0.42 and 0.43), the lowest mean value of the instrument assembly.

The mean scores for each dimension were as follows: *D1 = Awareness and regulation* (M = 2.81, SD = 0.36), D2 = Education and *teachers* (M = 2.81, SD = 0.31), *D3 = Regulation* and obligation (M = 1.47, SD = 0.59), and *D4 = Consequences* and risks (M = 1.20, SD = 0.41). The mean of the questionnaire as a whole was (M = 2.05, SD = 0.22).

As noted, in this work, we will focus on *Dimension 1 (Awareness and regulation)* with independent variables: gender, age, sector, HDI, and continent.

### 3.1. Gender Impact Analysis

In Table 4, the distribution of the sample according to the gender can be observed: *G0 = Woman or G1 = Man* for dimension D1.

As can be seen in Table 4, the distribution of the sample according to gender is uneven; the percentage of women (69.4%) is much higher than that of men (30.6%).

To analyze whether gender differences exist in the questionnaire, an ANOVA was performed for independent samples. The results are listed in Table 5.

Post-hoc tests suggest that the mean of women is above that of men in *dimension D1 = Awareness and regulation,* with higher means and propensity towards YES, so women could generally be inferred to have a higher awareness of vaccines than men.

Statistically significant differences appear in item P02 and dimension D1, although the size of the effect measured in the ANOVA test per eta squared being less than 0.06 has to be considered weak.

### 3.2. Age Incidence Analysis

In Table 6, the distribution of the sample by age group can be observed: E1 = Under 30, E2 = Between 30 and 44, E3 = Between 45 and 59, E4 = Greater than 60.

As can be seen in Table 6, the distribution of the sample by age group is uneven, the percentages of the age groups *E1 = Under 30* (36.3%), *E2 = Between 30 and 44* (34.8%), and *E3 = Between 45 and 59* (26.76%) are similar, while *the percentage of the group (E4) greater than 60* (2.2%) is of a lower order of magnitude.

To analyze whether there are differences according to the age group in the questionnaire, an ANOVA was performed for independent samples. The results are listed in Table 7.

Post hoc tests suggest that the mean age group under 30 is above the other age groups in dimension *D1 = Awareness and regulation*, with higher means and, therefore, propensity for YES, so it could be inferred that the age group under the age of 30, in general, has a higher awareness of vaccines than the rest of the age groups.

Statistically significant differences appear in items P01, P02, and dimension D1, although, as the size of the effect measured in the ANOVA test per eta squared was less than 0.06, it has to be considered weak.

### 3.3. Analysis of the Impact of the Sector

In Table 8, the distribution of the sample by sector can be observed: *S1 = Health, S2 = Education, S3 = Economy*.

As can be seen in Table 8, the distribution of the sample by sector is uneven; the percentage of the health sector (55.4%) is more than half of the sample, while the percentage of the education sector (32.9%) is one-third of the sample, and the share of the economy sector (11.7%) is a minority.

To analyze whether there are differences according to the sector in the questionnaire, an ANOVA was performed for independent samples. The results are listed in Table 9.

Post hoc evidence suggests that the mean education sector is above the health sector in dimension D1 = *Awareness* and regulation, with higher means and, therefore, with propensity towards YES, so it could be inferred that the education sector generally has a higher awareness of vaccines than the health sector, which has a more negative view. The means of the economy sector are in an intermediate position, presenting significant differences with the health sector and not significant with the education sector.

Statistically significant differences appear in items P01, P02, and dimension D1, although the size of the effect measured in the ANOVA test per eta squared when less than 0.06 has to be considered weak.

### 3.4. Impact Analysis by Human Development Index (HDI)

In Table 10, the distribution of the sample by Human Development Index (HDI) can be observed: *I1 = Very High, I2 = High, I3 = Medium, I4 = Low*.

As can be seen in Table 10, the sample distribution by Human Development Index (HDI) is uneven; the percentage of the group *I1 = Very high* (87.3%) is of an order of magnitude greater than the percentage of groups *I2 = High* (8.5%), *I3 = Medium* (3.1%), and *I4 = Low* (1.1%) that are of the same order of magnitude.

To analyze whether there are differences according to the Human Development Index (HDI) in the questionnaire, an ANOVA was conducted for independent samples. The results are listed in Table 11.

The post hoc tests suggest that the mean of the HDI group: *I1 = Very* high is above the other groups in *dimension D1 = Awareness and regulation*, with higher means and, therefore, with propensity towards YES, so it could be inferred that the HDI group: *I1 = Very high,* in general, has a higher awareness of vaccines than the rest of the groups.

Statistically significant differences appear in items P01, P02, and dimension D1, although the size of the effect measured in the ANOVA test per eta squared being less than 0.06 has to be considered weak in item P02, while item P01 and dimension D1, being eta squared above 0.06, can be considered with a mean effect.

### 3.5. Analysis of Incidence by Continent

Table 12 shows the distribution of the sample by continent: *C1 = Europe, C2 = America, C3 = Asia, C4 = Africa, C5 = Oceania*.

As can be seen in Table 12, the distribution of the sample by continent is uneven; the percentage *C1 = Europe* (83%) is of an order of magnitude greater than the percentage of the other *continents C2 = America* (9.3%), *C3 = Asia* (4%), *C4 = Africa* (3.5%), and *C5 = Oceania* (2%), which are of the same order of magnitude.

To analyze whether differences exist according to the continent in the questionnaire, an ANOVA was performed for independent samples, excluding Oceania analysis, as only two participants from that continent completed the questionnaire, which makes it unsuitable for statistical analysis using resampling techniques through the Monte Carlo simulation method using *the bootstrap algorithm*. The results are listed in Table 13.

In items P01, P02, and dimension D1, the mean of respondents from *the continent C1 = Europe* is above the rest of the continents in *dimension D1 = Awareness* and regulation, with higher means and, therefore, with propensity towards YES, so it could be inferred that respondents from the continent *C1 = Europe generally* have a higher awareness of vaccines than respondents from other continents.

Statistically significant differences appear in items P01, P02, and dimension D1, although the size of the effect measured in the ANOVA test per eta squared being less than 0.06 has to be considered weak in item P01, whereas, item P02, being eta squared above 0.06, can be considered with a mean effect.

## 4. Discussion and Conclusions

Research on the importance of vaccines in health is very broad, so the conclusions offered in this article are only a part of this. However, the results reflect a very interesting picture of the problem of vaccination from the world’s vaccine awareness and regulation dimension (D1) in a period closely linked to the start of the COVID-19 pandemic, since the completion of the work was in March 2020. 

It is important to note that 100% of respondents of all ages, sex, profession, human development index, and continent positively value vaccines as basic tools to ensure the health of citizens and, above all, to prevent possible contagions, stressing that, since vaccines, infant mortality has fallen considerably, and only in cases where vaccines are not yet available is there significant mortality, especially in developing countries. In this regard, it should be recalled that Article 35 of the Charter of Fundamental Rights of the European Union [35] provides that everyone has the right to health prevention and to benefit from the corresponding medical care in accordance with the conditions laid down in national provisions. Moreover, it is essential to protect the lives of infants, making it totally incomprehensible not to do so against diseases that can be avoided with vaccines.

Society’s awareness of the importance of vaccines and their relevant regulation by administrations has been an issue whose visibility in the media and social media has been increasing in the last decade, mainly due to their effectiveness and immersion of immunization around the world. However, they have also influenced vaccine movements with their constant public positioning against vaccination. Indeed, reluctance to vaccinate all countries is concerned not only with low-income countries, but has become a problem affecting everyone. That is why WHO [20] has repeatedly expressed concern at the expansion of anti-vaccine speakers, covering not only certain rural minorities, but even expanding in urban areas with high purchasing power, and in all social classes [36]. There is no doubt that raising awareness of the importance of vaccination is a key challenge that all international agencies must address in order to ensure human rights and peaceful coexistence in inclusive societies.

In the research carried out, it has been shown from a gender perspective that the mean of women makes a higher assessment than men in considering that society is aware of the importance of vaccines (D1, P1). However, there are no significant gender differences in whether administrations have regulated actions for vaccine compliance (D1, P2). This outcome could be affected by mothers who are mostly responsible for their sons’ and daughters’ vaccination schedule, so they could have a more up-to-date attitude, training, and information.

The group of people under the age of 30 is significantly different from the other age groups in their positive assessment of the awareness of society (D1, P1). In addition, higher scores are also evident in the group of people under 30 years of age, although of lower value, on whether administrations have regulated actions for the compliance of vaccination (D1, P2). There is no doubt that progress in vaccination over the past three decades has been very significant, not only because of technological advances, but also because of the increased incidence of vaccines, transparency, and safety.

Three main sectors have been considered: health, education, and economics, with the aim of studying whether there were differences on the subject of research. The mean of education sector has been shown to be significantly higher than that of the health sector, with the economy sector in an intermediate position. In this regard, other work shows that, in some areas, education contributes to greater acceptance and recognition of vaccination [37]. However, it was shocking to researchers that the health sector was the least valued at raising society’s awareness of the importance of vaccination and regulating administrations’ actions for vaccine compliance, so research would need to be done on the reasons for this assessment and its possible impact to improve social awareness campaigns.

In relation to the Human Development Index (HDI), it has been shown that the mean HDI = very high group is higher than that of the HDI = medium and low groups, offering results that are totally consistent with studies done by WHO.

It is the continent C1 = Europe in which we find significantly higher means compared to other continents (C2 = America; C3 = Asia; C4 = Africa; and C5 = Oceania).

In conclusion, it can be indicated that the profiles of women, people under 30 years of age, the education sector, high human development index, and the European continent are those that most values society’s awareness of the importance of vaccination (D1, P1) and the regulation of actions for the implementation of vaccination (D1, P2). It is a very hopeful conclusion to advance the construction of inclusive societies, creating greater involvement of society as a whole through effective and evidence-based communication to allay fears, address concerns, and promote acceptance of vaccination around the world. It should, therefore, be remembered that people who delay or reject vaccination for themselves or their children pose a growing challenge for countries seeking to close immunization gaps. According to WHO [12], one in five children worldwide do not yet receive regular vital immunizations, and around 1.5 million children die each year from diseases that could be prevented with vaccines that already exist.

The responsible awareness and active involvement of society worldwide about the importance of vaccination is critical to advancing the construction of a better world with safe planning and regulation that ensures compliance. Significantly, international agencies must be vigilant in order to deal with any incidents that may occur. Indeed, as has been shown in 2020, as a result of the COVID-19 pandemic, more than 14 million infants did not receive an initial dose of the DTP vaccine (diphtheria, tetanus and pertussis), demonstrating inadequate access to health and immunization services, and more than 5.7 million are only partially vaccinated. Similarly, more than 60% of these 19.7 million children live in 10 countries: Angola, Brazil, Ethiopia, the Philippines, India, Indonesia, Mexico, Nigeria, Pakistan, and the Democratic Republic of the Congo. In addition, non-vaccination is causing diseases that were considered eradicated, such as measles, to emerge, with new outbreaks active in the United States and in several countries in Europe (Portugal, Italy, Romania, etc.). It is clear that society’s awareness and proper regulation by administrations around the world will achieve collective immunity through safe and effective vaccines and make diseases rarer and, therefore, save lives.

In short, the research has highlighted the need for society to become aware of the importance of vaccination, so that clear, truthful, and relevant information that transcends safety in citizens about vaccination should be conveyed. In addition, they must report that infectious diseases are a major cause of morbidity and mortality across the globe, mainly in older people and those with chronic diseases. Consequently, effective and evidence-based communication is key to allaying fears, addressing concerns, and promoting acceptance of vaccination around the world. Reasonably, adequately raising awareness of the importance of vaccines will lead to the building of inclusive societies in which all citizens enjoy the health benefit, ensuring compliance with SDG3, “Ensuring healthy living and promoting well-being for all at all ages”, without discrimination of any kind.

## Figures and Tables

**Table 1:** Instrument structure.

Dimension	Items	α	CVI
D1 = Awareness and regulation	1, 2	0.52	0.87
D2 = Education and teachers	3, 4, 5, 6	0.56	0.93
D3 = Regulation and obligation	7, 8	0.57	1
D4 = Consequences and risks	9, 19, 11, 12	0.92	1
Mean		0.64	0.96

Source: own elaboration.

**Table 2:** Characteristics of the sample.

Sample Characteristics										
Gender	Women		Men							
	*n*	%	*n*	%						
	694	69.4%	306	30.6%						
Age	−30		30–44		45–59		+60			
	*n*	%	*n*	%	*n*	%	*n*	%		
	363	36.30%	348	34.80%	267	26.76%	22	2.20%		
IDH	Very High		High		Medium		Low			
	*n*	%	*n*	%	*n*	%	*n*	%		
	873	87.3%	85	8.5%	31	3.1%	11	1.1%		
Sector	Health		Education		Economy					
	*n*	%	*n*	%	*n*	%				
	554	55.4%	329	32.9%	117	11.7%				
Continent	Africa		America		Asia		Europe		Oceania	
	*n*	%	*n*	%	*n*	%	*n*	%	*n*	%
	35	3.5%	93	9.3%	40	4%	830	83%	2	0.2%

Source: own elaboration.

**Table 3:** Counting after the application of the questionnaire.

	Scale (*n*)					95% CI		95% CI
Item and Dimension (D)	1	2	3	*n*	M	Lower–Upper	SD	Lower–Upper
P01. Do you value that society is aware of the importance of vaccination?	26	174	800	1000	2.77	2.75–2.80	0.48	0.44–0.51
P02. Do you believe that administrations have regulated actions for vaccine compliance?	14	132	854	1000	2.84	2.81–2.86	0.40	0.37–0.44
D1 = Awareness and regulation					2.81	2.78–2.83	0.36	0.34–0.38
P03. Do you consider that, in your country, the training of compulsory education teachers (Children and Primary) provides adequate training on vaccines?	34	131	835	1000	2.80	2.77–2.83	0.48	0.44–0.52
P04. Do you think teachers are aware of the proper use of vaccines?	27	152	821	1000	2.79	2.77–2.82	0.47	0.43–0.50
P05. Do you think parents know the consequences that coexistence with other non-vaccinated peers could have on their children?	35	139	826	1000	2.79	2.76–2.82	0.49	0.44–0.52
P06. Do you believe that teachers at mandatory levels should receive initial training on health education and specifically on the vaccination process?	35	74	891	1000	2.86	2.83–2.88	0.44	0.40–0.49
D2 = Education and teachers					2.81	2.79–2.83	0.31	0.28–0.33
P07. Do you think it is necessary for teachers to require students to have a scheduled vaccination card?	616	183	201	1000	1.59	1.53–1.64	0.80	0.78–0.83
P08. Do you know if there is adequate regulations to support teachers in the demand to comply with childhood vaccination?	701	240	59	1000	1.36	1.32–1.40	0.59	0.56–0.62
D3 = Regulation and obligation					1.47	1.44–1.51	0.59	0.56–0.61
P09. Do you think teachers know the consequences that coexistence with non-vaccinated children could have on students and their person?	791	174	35	1000	1.24	1.21–1.28	0.50	0.47–0.54
P10. Do administrations have measures to ensure the health of pregnant teachers?	828	149	23	1000	1.20	1.17–1.22	0.45	0.41–0.48
P11. Do administrations have measures to ensure the health of teachers with minor children or family members with at-risk diseases?	840	143	17	1000	1.18	1.15–1.20	0.42	0.39–0.46
P12. Do parents of vaccinated students know the risk of their children when living with other non-vaccinated peers?	836	147	17	1000	1.18	1.16–1.21	0.43	0.39–0.46
D4 = Consequences and risks					1.20	1.18–1.23	0.41	0.37–0.44
Total					2.05	2.04–2.06	0.22	0.20–0.23

Source: own elaboration.

**Table 4:** Gender count of the participating sample for dimension D1.

		GENDER						
		Man			Woman			
Dimension (D)	Item	1	2	3	1	2	3	*n*
D1 = Awareness and regulation	P01	8	66	232	18	108	568	1000
	P02	5	55	246	9	77	608	1000

Source: own elaboration

**Table 5:** ANOVA for gender independent samples for dimension D1.

Dimension (D)	Item	M	Man	SD	95% CI	M	Woman	SD	95% CI	*p*	Eta2	Direction
D1 = Awareness and regulation	P01	2.73	2.68–2.79	0.50	0.44–0.55	2.79	2.76–2.83	0.47	0.42–0.51	0.06	0.00	W > M
	P02	2.79	2.74–2.84	0.45	0.39–0.50	2.86	2.83–2.89	0.38	0.34–0.42	0.01	0.01	W > M
	D1t	2.76	2.72–2.80	0.40	0.35–0.43	2.83	2.80–2.85	0.35	0.32–0.37	0.01	0.01	W > M

Source: own elaboration

**Table 6:** Count by age group of the participating sample for dimension D1.

		AGE												
		E1 = −30			E2 = 30–44			E3 = 45–59			E4 = +60			
Dimension (D)		1	2	3	1	2	3	1	2	3	1	2	3	*n*
D1 = Awareness and regulation	P01	9	36	318	6	88	254	11	46	210	0	4	18	1000
	P02	2	26	335	5	59	284	7	44	216	0	3	19	1000

Source: own elaboration.

**Table 7:** ANOVA for independent samples by age group (E1, E2) for dimension D1.

Item	M	E1 = −30	SD	95% CI	M	E2 = 30–44	SD	95% CI	*p*	Eta2	Direction
P01	2.85	2.81–2.89	0.42	0.35–0.49	2.71	2.66–2.76	0.49	0.44–0.53	0.00	0.02	E1 > E2. E3
P02	2.92	2.88–2.95	0.30	0.23–0.35	2.80	2.75–2.84	0.43	0.38–0.49	0.00	0.02	E1 > E2. E3
D1t	2.88	2.85–2.92	0.29	0.25–0.33	2.76	2.71–2.80	0.40	0.36–0.43	0.00	0.03	E1 > E2. E3
**Item**	**M**	**E3 = 45–59**	**D**	**95% CI**	**M**	**E4 = +60**	**SD**	**95% CI**	***p***	**Eta2**	**Direction**
P01	2.75	2.68–2.81	0.52	0.45–0.59	2.82	2.64–2.96	0.39	0.20–0.49	0.00	0.02	E1 > E2. E3
P02	2.78	2.72–2.84	0.47	0.40–0.54	2.86	2.70–3.00	0.35	0.00–0.47	0.00	0.02	E1 > E2. E3
D1t	2.76	2.71–2.81	0.39	0.35–0.43	2.84	2.71–2.95	0.28	0.15–0.38	0.00	0.03	E1 > E2. E3

Source: own elaboration.

**Table 8:** Count by sector of the participating sample for dimension D1.

		SECTOR									
		S1 = Health			S2 = Education			S3 = Economy			
Dimension (D)	Item	1	2	3	1	2	3	1	2	3	*n*
D1 = Awareness and regulation	P01	23	109	422	2	35	292	1	30	86	1000
	P02	13	91	450	0	24	305	1	17	99	1000

Source: own elaboration

**Table 9:** ANOVA for independent samples by sector for dimension D1.

Item	M	S1 = Health	SD	95% CI	*p*	Eta2	Direction
P01	2.72	2.67–2.76	0.53	0.49–0.58	0.00	0.02	S2 > S1.S3
P02	2.79	2.75–2.83	0.46	0.41–0.51	0.00	0.02	S2 > S1
D1t	2.75	2.72–2.79	0.40	0.37–0.43	0.00	0.04	S2 > S1.S3
**Item**	**M**	**S2 = Education**	**SD**	**95% CI**	***p***	**Eta2**	**Direction**
P01	2.88	2.84–2.92	0.34	0.28–0.40	0.00	0.02	S2 > S1.S3
P02	2.93	2.90–2.96	0.26	0.21–0.30	0.00	0.02	S2 > S1
D1t	2.90	2.88–2.93	0.25	0.21–0.29	0.00	0.04	S2 > S1.S3
**Item**	**M**	**S3 = Economy**	**SD**	**95% CI**	***p***	**Eta2**	**Direction**
P01	2.73	2.64–2.81	0.47	0.40–0.53	0.00	0.02	S2 > S1.S3
P02	2.84	2.76–2.90	0.39	0.30–0.47	0.00	0.02	S2 > S1
D1t	2.78	2.71–2.85	0.37	0.32–0.41	0.00	0.04	S2 > S1.S3

Source: own elaboration.

**Table 10:** Count by Human Development Index (HDI) for dimension D1.

		IDH												
		I1 = Very High			I2 = High			I3 = Medium			I4 = Low			
Dimension (D)		1	2	3	1	2	3	1	2	3	1	2	3	*n*
D1 = Awareness and regulation	P01	11	135	727	12	31	42	1	6	24	2	2	7	1000
	P02	8	97	768	2	30	53	3	2	26	1	3	7	1000

Source: own elaboration.

**Table 11:** ANOVA for independent samples by Human Development Index (HDI) for dimension D1.

Item	M	I1 = Very high	SD	95% CI	M	I2 = High	SD	95% CI	*p*	Eta2	Direction
P01	2.82	2.79–2.85	0.42	0.38–0.45	2.35	2.20–2.50	0.72	0.63–0.79	0.00	0.08	I1 > I3 > I4 > I2
P02	2.87	2.85–2.89	0.36	0.33–0.40	2.60	2.49–2.71	0.54	0.47–0.61	0.00	0.04	I1 > I3 > I2 > I4
D1t	2.85	2.82–2.87	0.33	0.30–0.35	2.48	2.38–2.57	0.47	0.43–0.50	0.00	0.09	I1 > I3 > I4 > I2
**Item**	**M**	**I3 = Medium**	**SD**	**95**% CI	**M**	**I4 = Low**	**SD**	**95% CI**	***p***	**Eta2**	**Direction**
P01	2.74	2.55−2.92	0.51	0.28–0.68	2.45	2.00–2.89	0.82	0.33–1.00	0.00	0.08	I1 > I3 > I4 > I2
P02	2.74	2.50–2.94	0.63	0.24–0.84	2.55	2.10–2.89	0.69	0.33–0.93	0.00	0.04	I1 > I3 > I2 > I4
D1t	2.74	2.57–2.89	0.44	0.28–0.55	2.50	2.23–2.75	0.45	0.26–0.52	0.00	0.09	I1 > I3 > I4 > I2

Source: own elaboration.

**Table 12:** Count by continent of the participating sample for dimension D1.

		CONTINENT															
		C1 = Europe			C2 = America			C3 = Asia			C4 = Africa			C5 = Oceania			
Dimension (D)		1	2	3	1	2	3	1	2	3	1	2	3	1	2	3	*n*
D1 = Awareness and regulation	P01	12	122	696	3	38	52	8	7	25	3	7	25	0	0	2	1000
	P02	5	88	737	3	31	59	1	6	33	5	7	23	0	0	2	1000

Source: own elaboration.

**Table 13:** ANOVA for independent samples by continent for dimension D1.

Item	M	C1 = Europe	SD	95% CI	M	C2 = America	SD	95% CI	*p*	Eta2	Direction
P01	2.82	2.80–2.85	0.42	0.38–0.45	2.53	2.41–2.64	0.56	0.49–0.63	0.00	0.06	C1 > C2. C3
P02	2.88	2.86–2.90	0.34	0.30–0.38	2.60	2.49–2.71	0.55	0.47–0.63	0.00	0.06	C1 > C2
D1t	2.85	2.83–2.87	0.32	0.29–0.34	2.56	2.47–2.66	0.47	0.43–0.51	0.00	0.08	C1 > C2. C3. C4
**Item**	**M**	**C3 = Asia**	**SD**	**95% CI**	**M**	**C4 = Africa**	**SD**	**95% CI**	***p***	**Eta2**	**Direction**
P01	2.82	2.80–2.85	0.42	0.38–0.45	2.53	2.41–2.64	0.56	0.49–0.63	0.00	0.06	C1 > C2. C3
P02	2.88	2.86–2.90	0.34	0.30–0.38	2.60	2.49–2.71	0.55	0.47–0.63	0.00	0.06	C1 > C2
D1t	2.85	2.83–2.87	0.32	0.29–0.34	2.56	2.47–2.66	0.47	0.43–0.51	0.00	0.08	C1 > C2. C3. C4

Source: own elaboration.

## Data Availability

Due to the anonymity and confidentiality of the data obtained, the authors have not reported any of the data obtained, the purpose of which is exclusively the development of this research.
